# Supplementary material for: Are carbon emissions trading and green financial instruments synergistic? -Comprehensive quantitative research based on content analysis
Source: PLoS One. 2024 Mar 7;19(3):e0298601. doi: 10.1371/journal.pone.0298601 (PMC10919601; doi:10.1371/journal.pone.0298601)
Supplement: S1 Data — (ZIP) [file pone.0298601.s001.zip › Data/地级市Z_F工作报告.zip!/╒π╜¡/╝╬╨╦╩╨/└┤╫╘╣1⁄2╓┌║┼í╛┬φ┐╦╩2╛▌═°í┐_2014.docx]

各位代表：

现在，我代表市人民政府向大会作工作报告，请予审议，并请市政协委员和其他列席人员提出意见。

2013年工作回顾

2013年，市人民政府认真贯彻党的十八大和十八届二中、三中全会精神，深入实施省委“八八战略”和干好“一三五”、实现“四翻番”的决策部署，全面落实市委七届五次、六次全会精神，牢牢把握稳中求进、转中求好的工作总基调，锐意进取、克难攻坚，较好地完成了市七届人大三次会议确定的主要目标任务。全市实现地区生产总值3147.7亿元，增长9.3%；完成财政总收入517.5亿元，其中公共财政预算收入282.3亿元，分别增长9.7%和9.5%；城镇居民人均可支配收入、农村居民人均纯收入分别为39087元和20556元，增长9.5%和10.3%。

（一）着力扩大有效需求，经济保持平稳较快增长。实施招商选资和浙商回归“一号工程”，引进世界500强公司、全球行业龙头企业和总投资1亿美元以上项目23个，实际利用外资22.1亿美元，浙商回归到位资金210亿元。实施扩大有效投资“三抓三保”专项行动，完成固定资产投资1910.2亿元，增长16.3%，其中民间投资占比达到62.9%。强化项目要素保障，新增建设用地3万亩，消化利用供而未用土地2万亩，金融机构本外币贷款余额增长12.3%。完成200个市级政府投资项目竣工验收。鼓励企业开拓市场，扩大自主品牌产品和服务贸易出口，实现进出口总额317.6亿美元，其中出口215.1亿美元，分别增长10.5%和9.8%。加快城市商圈和农村商贸流通网络建设，积极培育新兴消费热点，全社会消费品零售总额增长13.1%。

（二）着力调整产业结构，转型升级步伐明显加快。组织实施“机器换人”现代化技术改造，加快推进省光伏产业“五位一体”创新综合试点，完成工业技改投入563.4亿元，高新技术产业增加值占规模以上工业增加值比重提高2.7个百分点。开展工业企业综合绩效评价，市本级和海宁市率先试行差别化城镇土地使用税政策，全市腾退低效用地1.2万亩。加快民营企业“二次创业”，完成“个转企”6025家、“规下转规上”516家，新增百亿制造业企业1家。建筑业产值突破1000亿元。加快服务业集聚发展，新增省级服务业集聚示范区3个，现代物流、旅游休闲等服务业发展态势良好，嘉兴港完成货物吞吐量6605万吨，集装箱吞吐量突破100万标箱，全年接待国内外游客4725万人次。实施农业现代化三年行动计划，新建粮食生产功能区14.8万亩、省级现代农业园区22个，实现农业总产值259.7亿元，粮食生产保持稳定。

（三）着力推进改革创新，发展动力进一步增强。实施创新驱动发展战略，各级财政科技投入12.1亿元，增长15.3%。加快集聚创新资源，新增创新载体37家，遴选“创新嘉兴·精英引领计划”人才项目133个，引进各类高层次人才3489名。中电科技集团第三十六研究所杨小牛当选中国工程院院士。深化与中科院、清华大学等大院名校合作，组织实施省级以上重大科技专项23个，新列国家创新基金项目17个。新增发明专利授权量407件，被列为国家知识产权示范市。积极推进各项国家、省改革试点，嘉善县域科学发展示范点和海宁要素市场化配置综合配套改革试点进展顺利，平湖产业结构调整机制创新试点、滨海港产城统筹发展试验区建设全面启动。在全省率先实行行政审批层级一体化改革，下放市级审批事项457项。扎实推进农村集体资产产权制度改革，95%的村级经济合作社完成股份制改造。加快金融创新步伐，新设各类地方法人金融组织9家，新增直接融资170亿元。

（四）着力深化城乡区域统筹发展，现代化网络型田园城市建设加快推进。完善市域基础设施，开工建设杭平申线航道改造项目，秦山核电方家山扩建、钱江通道北接线一期等工程扎实推进，嘉绍跨江通道建成投运。制定促进市本级经济发展的政策意见，顺利完成湘家荡管理体制调整。加快城市有机更新步伐，全市启动有机更新1万亩，中心城区子城广场、南湖湖滨等区块征迁工作加快推进，完成房屋征收2118户。强力开展城市交通治堵，完成禾兴路、勤俭路等交通干道综合整治工程，优化公交线路33条，全市新增专用停车位1.1万个，主副中心城区实现公共自行车全覆盖。开展“三小车”专项整治，顺利完成市区编号残疾车退运。强力实施“三改一拆”，改造旧住宅区、旧厂房、城中村2320万平方米，拆除违法建筑1266万平方米。开展农村土地整治复垦，改造集聚农房1.7万户，复垦土地1.3万亩。开展12个市级小城市培育试点，海盐县与丹麦松德堡市建立中欧城镇化伙伴关系。深化美丽乡村建设，新增美丽乡村先进镇16个。组织开展接轨上海系列活动，积极参与“山海协作”，援藏援疆援青援川等对口支援工作进展顺利。

（五）着力推进水环境综合整治，生态建设取得积极成效。全面落实市、县、镇三级“河长制”，市域范围1.3万公里河道实现责任全覆盖。加快推进截污纳管，新建污水收集管网393公里，2218家工业企业实现污水达标入网。认真贯彻省人大常委会决定，制定实施加强畜禽养殖污染防治促进畜牧业转型升级的政策意见，科学划定禁、限养区，加强日常监管，拆除违建猪舍579万平方米，生猪存栏量比年初下降了近三分之一。加强饮用水源保护，贯泾港、长水塘等生态湿地工程建设加快推进。实施千岛湖、太湖“双水源”战略，组织开展太湖引水项目书编制等工作。全市及各县（市、区）跨行政区域河流交接断面水质年度考核均为合格以上，其中嘉善、海盐、海宁、桐乡为良好。深入开展“清洁空气”专项行动，推进燃煤锅炉脱硫脱硝设施建设，新建改建空气监测站14个。严格执行能源消费总量和能耗强度双控制度，实施印染、造纸、化工、制革四大行业整治，否决高污染、高能耗项目161个，查处环境违法案件1712起，关停企业169家。大力实施“双清”和“四边三化”行动，清淤疏浚河道1086公里，新增和改造提升绿化面积4.5万亩。预计省下达的节能减排各项指标全面完成。

（六）着力保障和改善民生，社会保持和谐稳定。各级财政用于民生支出230.2亿元，占公共财政预算支出的75.9%。加强就业帮扶工作，新增城镇就业10.4万人，城镇登记失业率为2.9%。加快社会保险扩面提标，实施城乡居民合作医疗保险和职工基本医疗保险整合。健全社会救助机制，发放医疗救助金4618万元，开工建设保障性住房8086套，城乡居民最低生活保障标准分别提高到525元和384元。加强社会养老服务体系建设，新增居家养老服务照料中心273家、养老机构床位3340张。积极开展教育现代化县（市、区）创建，我市成为省内唯一所有县（市、区）全部通过国家义务教育均衡发展评估的地市。深化公立医院综合改革，做好人感染H7N9禽流感疫情防治工作，实现省级卫生强市、卫生强县（市、区）“满堂红”。积极创建国家公共文化服务体系示范区，城乡一体化公共图书馆服务体系通过国家首批示范项目验收。巩固提升全国文明城市创建成果，城市文明程度指数测评位列全省第一、全国第十。举办首届市民运动会，嘉兴籍运动员在第十二届全运会上取得了17枚奖牌的优异成绩。人口计生和新居民工作得到加强，工会、青少年、妇女、慈善、残疾人等事业全面发展，统计、档案、史志、气象等在服务经济社会转型发展中发挥了积极作用。全力抗击历史罕见高温干旱，有效保障生活生产用水。全力抗击“菲特”台风及其带来的有水文记录以来最大洪涝灾害，扎实做好受灾群众救助、灾区排涝防疫、灾后恢复生产等工作，实现了“不死人、少伤人、少损失、保安全”的目标。加强社会应急联动体系建设，认真做好信访工作，严厉打击各类违法犯罪活动，再次荣获全国社会管理综合治理优秀城市。大力支持国防和军队建设，扎实推进国防动员、双拥优抚等工作，成功承办“浙江金盾-13”人民防空应急应战救援演习。民族、宗教关系融洽和谐，外事、对台、侨务、港澳等工作取得新成效。

政府自身建设得到进一步加强。严格依法履行政府职责，主动向市人大报告工作、向市政协通报重大事项，办理市人大代表建议316件、市政协提案384件。落实行政规范性文件“三统一”制度，清理规范性文件114件，办结行政复议案件110起。深入开展走访服务和破解难题专项行动，积极帮助企业和基层解决实际困难。严格执行中央“八项规定”、省委“六项禁令”和“28条办法”，加强预算绩效管理，公务用车、公务接待、因公出国(境)支出下降15.7%。认真落实党风廉政建设责任制和廉政准则，大力推进廉政风险防控机制建设，腐败案件查办力度不断加大。

各位代表，过去一年的成绩来之不易，这是省委、省政府和中共嘉兴市委正确领导的结果，是全市上下同心同德、奋力拼搏的结果。在此，我代表市人民政府，向在各个岗位上辛勤劳动的全市人民，向给予政府工作大力支持的人大代表和政协委员，向各民主党派、工商联、人民团体和社会各界人士，致以崇高的敬意！向驻嘉人民解放军和武警官兵，向所有关心支持嘉兴发展的海内外朋友们，表示衷心的感谢！

我们也清醒地看到，我市经济社会发展中还存在不少困难和问题：经济稳中回升的基础还不稳固，结构性、素质性矛盾依然突出，科技创新能力有待提升，投入产出比偏低，中心城市辐射带动力不强，治水治气、节能减排等生态建设任务十分艰巨；城乡居民持续增收难度加大，城镇居民人均可支配收入增速与年初确定的目标尚有一定差距，事关群众切身利益的教育、医疗、安全生产、食品安全等方面还面临不少问题；政府职能转变仍然滞后，形式主义、官僚主义、享乐主义和奢靡之风等问题在一些地方和部门依然存在。对此，我们一定高度重视，采取有力措施认真加以解决。

2014年目标任务和主要工作

2014年是深入贯彻党的十八届三中全会精神、全面深化改革的第一年，也是落实“十二五”规划的关键一年。根据市委七届七次全体（扩大）会议精神，今年政府工作的指导思想是：认真贯彻党的十八届三中全会和中央有关会议精神，深入实施省委“八八战略”和干好“一三五”、实现“四翻番”的决策部署，牢牢把握科学发展主题，以加快转变经济发展方式为主线，以改革统揽全局，坚持稳中求进、改中求活、转中求好，着力在提质增效上见成效，在改善民生上出实招，在狠抓落实上下功夫，实现全面深化改革的良好开局，推动经济持续健康发展与社会和谐稳定，为加快“三城一市”和“两富”现代化建设奠定更加坚实的基础。

在政府工作中，要牢牢把握“五个更加注重”：更加注重深化改革。大力营造敢为人先、勇于创新、宽容失败的改革氛围，充分发挥经济体制改革牵引作用，正确处理好政府与市场、政府与社会的关系，加快推进制约经济社会发展的重点领域和关键环节改革，不断激发市场主体活力。更加注重发展质量效益。不简单以地区生产总值及增长率论英雄，更加突出人均生产总值、亩均税收、全员劳动生产率等衡量标准，加大约束性指标考核权重，坚定不移推进科技创新和产业升级，加快完善城乡发展一体化体制机制，努力实现有质量、有效益、可持续的发展。更加注重生态文明。坚决守住生态环境底线，实行最严格的环境保护制度和政策，坚持经济发展与环境保护并重、资源开发与节约集约利用并重，促进生态环境加快好转，努力让嘉兴的天更蓝、水更清、地更绿、空气更清新。更加注重民生改善。坚持尽力而为、量力而行，把新增财力、公共资源更多向民生倾斜，加快推进基本公共服务均等化，优先办好各项民生实事，努力为城乡居民创造数量更多、收入更高的就业岗位，实现居民收入增长与经济发展同步。更加注重平安建设。加强社会治理创新，健全群众诉求表达、利益协调、权益保障和社会矛盾化解机制，完善社会公共安全治理体系，确保人民安居乐业、社会安定有序，着力打造长三角最具安全感的城市。

综合考虑经济社会发展趋势和工作导向，建议2014年全市经济社会发展的主要预期目标为：地区生产总值增长8.5%；城镇居民人均可支配收入增长9.5%，农村居民人均纯收入增长10%；公共财政预算收入增长8%；研究与试验发展经费支出占生产总值比重2.5%；居民消费价格指数与省价格调控目标保持衔接；节能减排各项指标完成或超额完成省下达的目标任务。

围绕上述目标要求，今年市政府重点抓好以下六个方面工作：

（一）坚持以改革为统领，不断激发发展活力

加快政府自身改革。建立权力清单制度，依法削减行政权力，公开权力运行流程。深化行政审批层级一体化改革，加强行政审批事项下放运行管理，创新事中事后监管为主的管理机制。推进模拟制、代办制、联审制“三制联动”审批，构建全市统一的网上审批平台。建立负面清单制度，实施企业投资“零审批”试点。开展新一轮政府机构改革，推进政府职能转变。加快综合执法体制改革，提高执法和服务水平。加快事业单位分类改革，加大政府购买公共服务力度。积极培育社会组织，规范中介服务市场秩序。

推进要素配置市场化改革。强化“亩产论英雄”导向，扩大差别化城镇土地使用税试点范围，加快建立存量土地盘活、新增建设用地指标分配与土地产出效益挂钩制度，健全低效用地退出机制，力争全市腾退低效用地1万亩。推进水电气、建设用地、环境容量等资源要素差别化定价，建立健全工业用地、节能量、水权、排污权交易制度。加快金融创新发展，优化金融生态环境，发展普惠金融，支持嘉兴银行等地方性法人金融机构在服务地方经济社会发展中做大做强。

提升发展民营经济。坚定不移地把提升发展民营经济放在更加突出位置，取消和废止制约民间投资发展的各种歧视性政策规定。推进工商注册登记制度改革，抓好“个转企、小升规、规改股、股上市”工作，新增工商法人企业6000家以上、“规下转规上”500家。引导民营企业加强制度创新、管理创新和科技创新，支持民营企业参与国有企业改革重组。大力鼓励民间资本投向教育科研、医疗卫生、文化体育等社会事业和市政公用事业。

加快财政、国资改革步伐。完善财政管理体制，加快建立公开、透明、规范的预算制度，推进政府专项资金竞争性分配，切实提高财政资金绩效。健全税收征管体制，完善政府非税收入征收管理制度。严格政府举债程序，控制优化债务规模和结构，健全政府债务风险管控机制。推行国有企业分类管理，完善法人治理结构。以管资本为主加强国资监管，加快建立以效益、净资产收益率和社会贡献率为主要指标的分类考核评价体系。妥善处理市属企业改制历史遗留问题。

统筹推进各项改革试点。积极推进国家、省改革试点，用足用好先行先试政策，着力形成嘉兴改革的集群优势。加快嘉善县域科学发展示范点、海宁要素市场化配置综合配套改革试点和平湖产业结构调整机制创新试点建设，及时推广成功经验。支持桐乡全国旅游综合改革试点、海盐农村综合改革示范试点建设，不断积累经验。抓好滨海港产城统筹发展试验区建设，积极推进国家信息消费试点城市建设。

以开放促改革。充分发挥好嘉兴在推进长三角区域一体化发展中的“桥头堡”作用，深入开展接轨上海系列活动，进一步加强与上海的全方位对接。主动承接上海自贸区的辐射和溢出效应，抢抓机遇全力建设改革发展配套区。深化嘉兴港与周边大港合作，推动嘉兴出口加工区整合提升为国家级综合保税区，力争实现嘉兴港全域开放。加快高附加值产品和服务贸易出口，鼓励优势企业走出去投资建立经贸合作区、制造基地和市场营销网络，进出口总额增长6%。积极参与杭州都市圈和浙东经济合作区合作共建，扎实做好新一轮援藏援疆援青援川等对口支援工作。

（二）强力推进“五水共治”，加快建设美丽嘉兴

打好治水攻坚战。举全市之力治污水、防洪水、排涝水、保供水、抓节水，倒逼经济转型升级。完善“河长制”，坚决整治臭河、黑河、垃圾河，确保年底前基本消灭垃圾河，有效改善臭河、黑河水质。加大截污纳管力度，新建污水收集管网220公里，加快推进工业企业污水全入网，对无法按期入网企业实行分类整治。强化畜禽养殖污染治理，加快分散式污水处理设施建设，推进农村污水处理自然村全覆盖。完善区域联动治理机制，确保跨行政区域河流交接断面水质年度考核全部合格以上。实施水利建设“三年行动计划”，加快建设平湖塘延伸拓浚、扩大杭嘉湖南排等工程，清淤疏浚河道1000公里、建设标准圩区35万亩。实施最严格的水资源管理制度，开工建设一批应急备用水源项目，确保饮用水源安全。全力推进市域外引水工程，深化千岛湖引水工程前期工作，完成太湖引水工程立项批复并开展可研报告编制，力争项目早日开工。深入开展国家节水型城市建设，积极推广应用节水新技术、新工艺、新设备。

狠抓大气污染防治。大力调整能源结构，增加清洁能源供应量，削减煤炭消费总量。开展工业废气治理，积极推行“煤改气”，加快燃煤锅炉淘汰步伐。强化机动车尾气整治，做好公交车、出租车“油改气”工作，加快“黄标车”淘汰进度。建立健全禁止秸秆焚烧的长效监管机制，深入开展工地扬尘、餐厨油烟等专项整治，切实加强灰霾天气监测预警和治理。加大环保执法力度，强化重点区域、行业和企业的监管和综合整治。

联动推进城乡共治。进一步加大“三改一拆”力度，建立健全有违必拆的长效机制，全面开展无违建县（市、区）、无违建镇（街道）创建活动。积极开展以城乡垃圾清理为重点的清洁家园专项行动，完善城乡环境卫生保洁长效机制。加强固体废弃物管理，加大土壤污染治理力度。深入开展“四边三化”行动，加快推进城市楔形绿地以及城市出入口、河道两岸等绿化工程，新增和改造提升绿化面积4万亩。

（三）全力扩大有效投资，切实增强经济发展后劲

强化招商选资和浙商回归。坚持引浙商、招央企和选外资三管齐下，深入实施招商选资和浙商回归“一号工程”，确保实际利用外资15亿美元、浙商回归到位资金230亿元。坚持招大引强选优，力争引进世界500强公司、全球行业龙头企业和总投资1亿美元以上项目20个，每个国家级经济技术开发区至少引进一个总投资50亿元以上大项目，每个省级经济开发区至少引进一个总投资20亿元以上大项目。完善项目准入机制，坚决杜绝引进高污染、高能耗、高排放和低效益项目。

加快重大项目建设。把重大项目建设作为经济转型升级的重要抓手，确保固定资产投资增长13%。全年安排“三个千亿”项目488个，计划投资540亿元，加快建设杭平申线航道改造、桐九公路改建等一批工程，力争开工建设杭州湾跨海大桥北接线二期等项目，继续深化市域轨道交通、嘉兴军民合用机场等项目前期。完成市级政府性投资项目竣工验收150个以上。加强项目用地保障，积极争取更多项目列入国家、省重点项目计划获取点供指标，抓好批而未供、供而未用土地消化利用，全年盘活存量土地6500亩以上。

优化投资结构。以提高投资质量效益为导向，切实发挥好政府投资的带动作用，大力引导民间资本投向“五水共治”、科技创新和社会民生等领域，进一步提高民间投资比重。狠抓实体经济不放松，完善产业发展规划，出台优先扶持产业目录和配套政策，加快推进一批技术含量高、经济效益好的产业升级项目，力争工业投资突破1000亿元。深入实施服务业“百项百亿”工程，进一步提高服务业在固定资产投资中的比重。

（四）加快结构调整步伐，着力打造嘉兴经济升级版

实施创新驱动发展战略。修编嘉兴科技城规划，大力引进各类创新资源，探索开发建设新机制，全力打造科研与产业相融合的创新大平台。加快浙江科技孵化城（光伏高新园区）建设步伐，积极争创国家级高新技术产业园区。深化产学研合作，支持浙江清华长三角研究院、浙江中科院应用技术研究院加快发展，推进浙江大学国际联合学院等创新载体建设，筹建嘉兴科技成果转化交易中心。加强镇（街道）科技服务中心建设。大力引进各类风险投资机构，扎实推进省科技金融改革创新试验区建设。强化企业创新主体地位，积极扶持成长性科技型中小企业，新增省级企业技术（研发）中心30家以上。深入实施“创新嘉兴·精英引领计划”，完善柔性引才工作机制，新建院士专家工作站5家以上，年内引育领军人才100名左右，实现国家“千人计划”县（市、区）全覆盖。

大力发展先进制造业。以“四换三名”工程为抓手，深化工业强市建设。加快“腾笼换鸟”步伐，深入实施印染、造纸、化工、制革四大行业整治，力争提前一年完成“十二五”落后产能淘汰目标任务。加快推进省光伏产业“五位一体”创新综合试点和中国核电城建设，大力发展高端装备制造、高端新材料、新一代电子信息和新型节能环保等产业，力争战略性新兴产业产值突破2800亿元。深入实施“两化”融合，加快改造提升传统产业，全面推进“机器换人”，实施技术改造项目1000个。积极推进“空间换地”，促进开发区、工业园区集约发展、提质增效，省级以上经济开发区加快建设成为高新技术园区，加大中小企业创业中心和小微企业园建设力度，新建标准厂房50万平方米。大力推行“电商换市”，鼓励实体企业发展电子商务，积极创建国家电子商务示范城市。深化质量强市建设，大力培育名企名品名家，年内新增百亿制造业企业2家、浙江名牌12个，积极培育壮大高素质企业家队伍和高技能人才队伍。

优先发展服务业。加快国际商务区核心区建设步伐，扎实推进服务业集聚区建设，着力引进一批重点项目和企业，大力培育总部经济、研发设计、物联网、商务会展等高端业态。优化市域物流园区布局，鼓励专业市场创建区域品牌和集体商标，加快推进南湖区基金小镇、国际商务区金融广场建设，推动现代物流、专业市场、金融服务等生产性服务业提速提质发展。完善城乡消费网络，大力发展商贸流通、健康养老、信息消费等生活性服务业，全社会消费品零售总额增长12%。加强旅游资源整合优化，提升旅游公共服务平台功能，着力打响大嘉兴旅游品牌。

加快农业现代化进程。深化农业“两区”建设，新建粮食生产功能区14万亩。加快培育新型农业经营主体，大力发展现代种子种苗产业，积极推广“万元千斤”等新型生态循环种植模式。严格落实生猪养殖总量削减计划，坚决拆除违建猪舍，引导规模化、生态化养殖，促进生猪养殖业减量提质。加强农业“三位一体”公共服务体系建设，扩大农业保险范围，进一步发挥好农合联作用。支持农科院等机构加强农业科技创新。强化农民职业技能培训，组建农民学院。

（五）统筹推进新型城市化和新农村建设，努力实现更高水平的城乡一体化

优化市域空间布局。坚持规划“一套图”和空间“全覆盖”，修编完善市域总体规划和城市总体规划，与土地利用总体规划、产业发展规划相衔接，加快市域融合发展步伐，促进县域经济向城市经济转型。完善规划管理体制，强化规划执行刚性。制定落实促进市本级经济发展的具体措施，加快推进国际商务区、湘家荡区域、温泉新城等重点区块开发建设，完善城市功能，着力提高中心城市首位度。支持各副中心城市特色发展，加快形成错位发展的新格局。加大中心镇培育力度，深化省、市两级小城市培育试点工作。完善耕地保护共同责任机制，科学设置土地开发强度，提高城镇建设用地利用效率和集约化水平，不断优化城乡建设用地空间。

提升城市建设管理水平。加大城市有机更新力度，加快推进中心城区子城广场项目建设，统筹抓好各重点区块改造，全市启动有机更新1万亩。优化城市交通拥堵治理，加强城市综合交通规划研究，实施“增支路、连断点、疏瓶颈、建场站”工程，新建改建三环南路、三元路等道路，打通由拳路、长水路等一批断头路，科学规划公共停车场所，完善交通微循环管理，市区新增专用停车位5000个，全市建成地下空间130万平方米以上。坚持公交优先发展，整合优化公交线路，提高公交班次密度，市区公交分担率提高2个百分点。完成市区四（三）轮电动车整治和人力客运三轮车退运工作。积极推进“智慧城市”建设，着力提高城市管理的科学化、精细化水平。

加快新农村建设步伐。落实农民对承包地占有、使用、收益及承包经营权抵押、担保权能，规范土地承包经营权有序流转，新增流转面积8万亩以上。深入做好宅基地确权登记工作，探索建立农村宅基地有偿退出和跨社置换机制，稳步推进农民住房财产权抵押、担保、转让试点。深化农村集体资产产权制度改革，健全农村产权交易平台，开展农村集体资产股权流转试点，发展壮大村级集体经济。深化户籍制度改革，促进有能力在城镇就业和生活的农村居民有序实现市民化。调整优化村庄布局，稳步推进农村土地整治，强化农房设计服务，逐步解决农民刚性建房需求，年内完成土地整治复垦1万亩。深化美丽乡村建设，深入推进“百村示范、千村整治”工程，加快城市基础设施、公共服务向农村延伸，高标准建成一批省重点培育示范中心村，加强历史文化村落保护，进一步彰显江南水乡特色。

（六）加强社会建设，着力增进民生福祉

健全就业创业和社会保障体系。完善公共就业服务机制，规范发展农村劳务合作社，加大公益性岗位开发力度，新增城镇就业岗位6.5万个，城镇登记失业率控制在4%以内。加强创业培训服务，实施大学生创业圆梦行动，积极创建国家创业型城市。继续推进城乡居民基本医疗保险和职工基本医疗保险整合工作，建立健全社会保障待遇确定和正常调整机制。完善城乡一体的社会救助体系，加快城乡居民最低生活保障标准并轨步伐。积极应对人口老龄化，深化城乡养老服务体系建设，大力鼓励社会资本参与养老产业发展，积极推进为低收入群众服务的养老机构建设，居家养老服务照料中心覆盖80%以上的城市社区、50%以上的农村社区。加大公共租赁房建设力度，开工建设保障性住房4181套。积极发展红十字会和公益慈善事业，加强残疾人社会保障和服务体系建设，维护妇女儿童和未成年人合法权益。

大力发展社会事业。培育和践行社会主义核心价值观，完善城乡精神文明建设长效机制，确保成功蝉联全国文明城市。积极开展国家公共文化服务体系示范区创建，完善“文化有约”服务平台，推进优质文化资源更多向基层、向农村倾斜。加快文化产业集聚区建设，着力培育一批重大文化产业项目和品牌。加强文化遗产保护和利用。加快教育现代化步伐，实施第二轮学前教育“三年行动计划”，扎实推进义务教育标准化学校创建，加强职业教育校企合作联盟建设，促进高等教育内涵式发展。重视名师名校长培养，加大公办学校教师校长交流轮岗力度，切实加强师德师风建设。开展多种形式的科普活动，提高全民科学素养。推进“健康嘉兴”建设，深化公立医院综合改革，健全基层医疗卫生机构运行机制，积极推进商业医疗保险，实现国家卫生城市创建县（市、区）“满堂红”。实施“单独两孩”政策。推进公共体育设施免费开放，积极创建省体育强市。支持工会、共青团、妇联、科协等群团组织发展和参与社会治理，做好民族宗教、外事侨务、对台事务等工作。

深化“平安嘉兴”建设。推动社区、社团和社工互动发展，加强新居民管理服务，夯实社会管理基层基础。深化安全管理体制改革，建立隐患排查和安全预防控制长效机制，促进安全生产形势持续稳定好转。创新食品药品和农产品安全监管体制机制，建立健全全过程监管和可追溯制度。加强企业诚信制度建设，完善信用约束机制。强化网络社会管理，促进网络依法规范有序运行。深化社会普法教育，加强法律服务和法律援助。健全重大决策社会稳定风险评估机制，深入开展领导干部接访下访活动，做好人民调解工作。推进应急平台体系和防灾减灾工作规范化建设。完善立体化社会治安防控体系，严密防范和依法严厉打击违法犯罪活动。大力支持驻嘉人民解放军和武警部队建设，加强国防动员、民兵预备役和人民防空工作，促进军政军民团结，实现省双拥模范城市“六连冠”。

全面加强政府自身建设

新形势、新任务对政府工作提出了更高要求，我们必须坚持为民、务实、清廉，做到政府工作为人民服务、对人民负责、受人民监督、让人民满意，绝不辜负全市人民的期待和重托。

着力建设为民政府。始终保持与人民群众的血肉联系，扎实开展党的群众路线教育实践活动，全面贯彻落实中央和省改进工作作风、密切联系群众的各项规定，带着感情见群众，带着问题下基层。坚持问政于民、问计于民、问需于民，凡是涉及群众利益的决策都要充分听取群众意见，凡是损害群众利益的做法都要坚决防止和纠正。始终把人民群众的期盼作为工作的取向和动力，勤勤恳恳、想方设法为群众多做贴心事、实在事，今年市政府将实施强化污水治理、整治大气污染、建设城乡社区居家养老服务照料中心等10项民生实事项目。

着力建设效能政府。坚持“实干论英雄”，大力弘扬勇于担当、敢于创新的优良作风，满怀激情干事创业，真抓实干破解难题，比学赶超、争创一流。加强调查研究，精简会议和文件，严控各类庆典表彰和评比达标活动，坚决反对做表面文章、搞形式主义。牢固树立“一盘棋”思想，调动各方力量，合理配置行政资源，确保政令畅通。深化绩效管理，完善考评机制，加强督查督办，严肃查处推诿扯皮、行政不作为等行为，进一步提升政府的执行力、公信力。

着力建设法治政府。坚持依法行政，认真落实市人大及其常委会各项决议决定，自觉接受人大法律监督、工作监督和政协民主监督，认真办理人大代表建议和政协提案。积极听取民主党派、人民团体和各界人士的意见建议，主动接受司法监督和舆论监督。完善重大事项和重大项目科学民主决策机制，加强新型智库建设，健全决策咨询制度。规范行政执法裁量权，加强行政复议规范化建设。全面实行政务公开，深化电子政务工作，完善网络问政平台，保障落实公民的知情权、参与权、表达权和监督权，让权力在阳光下运行。

着力建设廉洁政府。认真贯彻中央关于厉行节约反对浪费各项规定和国务院“约法三章”，严格执行公车配备使用管理、办公用房、公务接待、因公出国（境）等方面的具体制度，“三公”经费在2013年预算的基础上下降30%以上。严格落实党风廉政建设责任制，强化行政监察和审计监督，切实加强对规划审批、项目招投标、土地出让、政府采购、国有产权交易、财政资金补助等重点领域和关键环节监管，严肃查处各类违纪违法案件，坚决防止和纠正损害群众利益的不正之风。

各位代表，嘉兴正处在深化改革、转型发展的关键时期，我们的任务艰巨而繁重，责任重大而光荣。让我们更加紧密地团结在以习近平同志为总书记的党中央周围，在省委、省政府和中共嘉兴市委的正确领导下，紧紧依靠全市人民，开拓进取，真抓实干，为加快推进“三城一市”和“两富”现代化建设而努力奋斗！
